# Supplementary material for: Direct and Ultrasensitive Bioluminescent Detection of Intact Respiratory Viruses
Source: ACS Sens. 2024 Oct 8;9(10):5550–60. doi: 10.1021/acssensors.4c01855 (PMC11519905; doi:10.1021/acssensors.4c01855)
Supplement: Supplementary file 1 — se4c01855_si_001.pdf [file se4c01855_si_001.pdf]

Direct and ultrasensitive bioluminescent detection of intact respiratory viruses

Alexander Gräwe[1], Harm van der Veer[1], Seino A.K. Jongkees[2], Jacky Flipse[3,4], Iebe Rossey[5], Robert P. de Vries[6], Xavier Saelens[5], Maarten Merkx[1*]

1 Laboratory of Protein Engineering, Department of Biomedical Engineering and Institute for Complex Molecular Systems, Eindhoven University of Technology, Eindhoven, The Netherlands. E-mail: m.merkx@tue.nl
2 Department of Chemistry and Pharmaceutical Sciences, Amsterdam Institute of Molecular and Life Sciences, Vrije Universiteit Amsterdam, Amsterdam, The Netherlands
3 Laboratory for Medical Microbiology and Immunology, Rijnstate Hospital, Arnhem, The Netherlands
4 Laboratory for Medical Microbiology and Immunology, Dicoon, Elst, the Netherlands
5 VIB Center for Medical Biotechnology, Department of Biochemistry and Microbiology, Ghent University, Ghent, Belgium
6 Utrecht Institute for Pharmaceutical Sciences, Department of Chemical Biology and Drug Discovery, Utrecht, The Netherlands

*Corresponding author: Maarten Merkx, m.merkx@tue.nl

Supporting Information

This PDF file includes:

Figures S1 to S12
Section S1 Supplementary Materials and Methods
Section S2 Protein Sequences

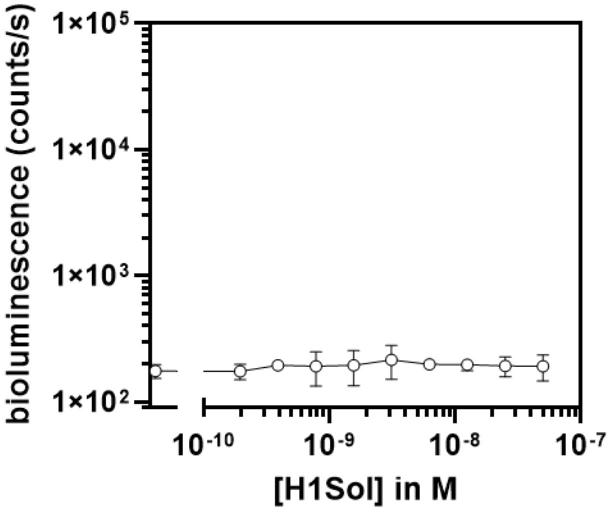

Figure S1.

GLOVID without binders attached.

LgBiT-Dog1 and SmBiT-Dog1 were mixed and used in titrations agains soluble, trimeric viral surface protein H1Sol (H1 from A/Solomon Islands/3/2006 (H1N1). Experimental conditions: 5 nM LgBiT-Dog1, 5 nM SmBiT-Dog1, final NanoGlo dilution 1:1000, 1 h incubation 22 °C. Error bars represent the standard deviation of n=3 replicates.

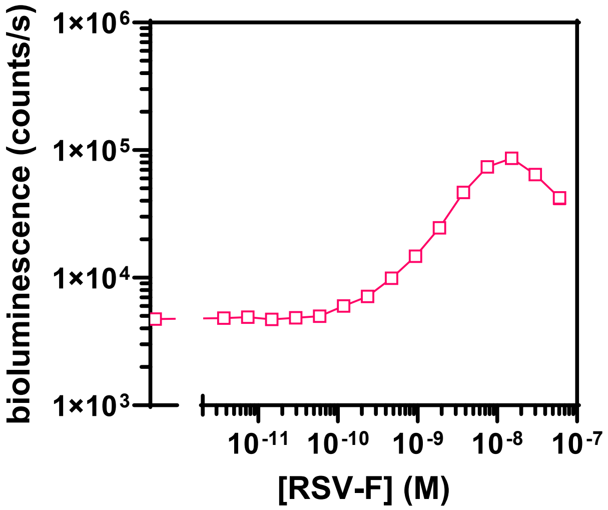

Figure S2.

Trivalent GLOVID with F-VHH-4.

GLOVID with anti-prefusion RSV-F nanobody F-VHH-4 conjugated to LgBiT-Dog3 and SmBiT-Dog3 (trivalent system). Experimental conditions: 1 nM GLOVID components, 1xPBS + 1mg/ml BSA, final NanoGlo dilution 1:2000, 1 h incubation 22 °C. The mean of n=3 replicates is shown, with error bars too small to be depicted.

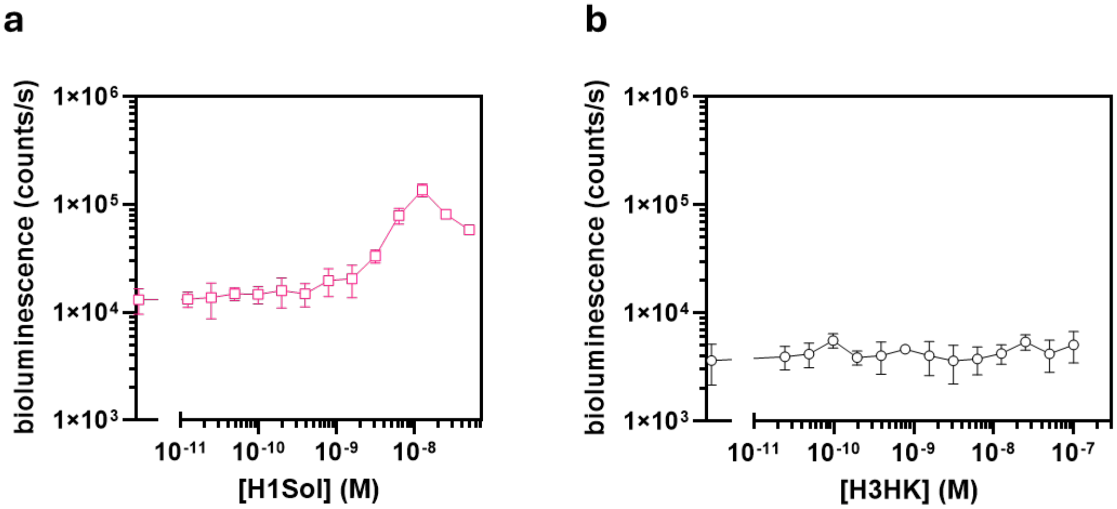

Figure S3.

Trivalent GLOVID and control on H3HK with S5.

a) Trivalent GLOVID where cyclic peptide S5 was conjugated to each LgBiT-Dog3 and SmBiT-Dog3 and used in titrations of H1Sol. b) Control GLOVID on H3HK with cyclic peptide S5 conjugated to both sensor components. Experimental conditions: 2 nM LgBiT, 6 nM SmBiT, 1xPBS, 1 h incubation at 22 °C, 1:1000 diluted NanoGlo. Error bars represent the standard deviation of n=3 replicates.

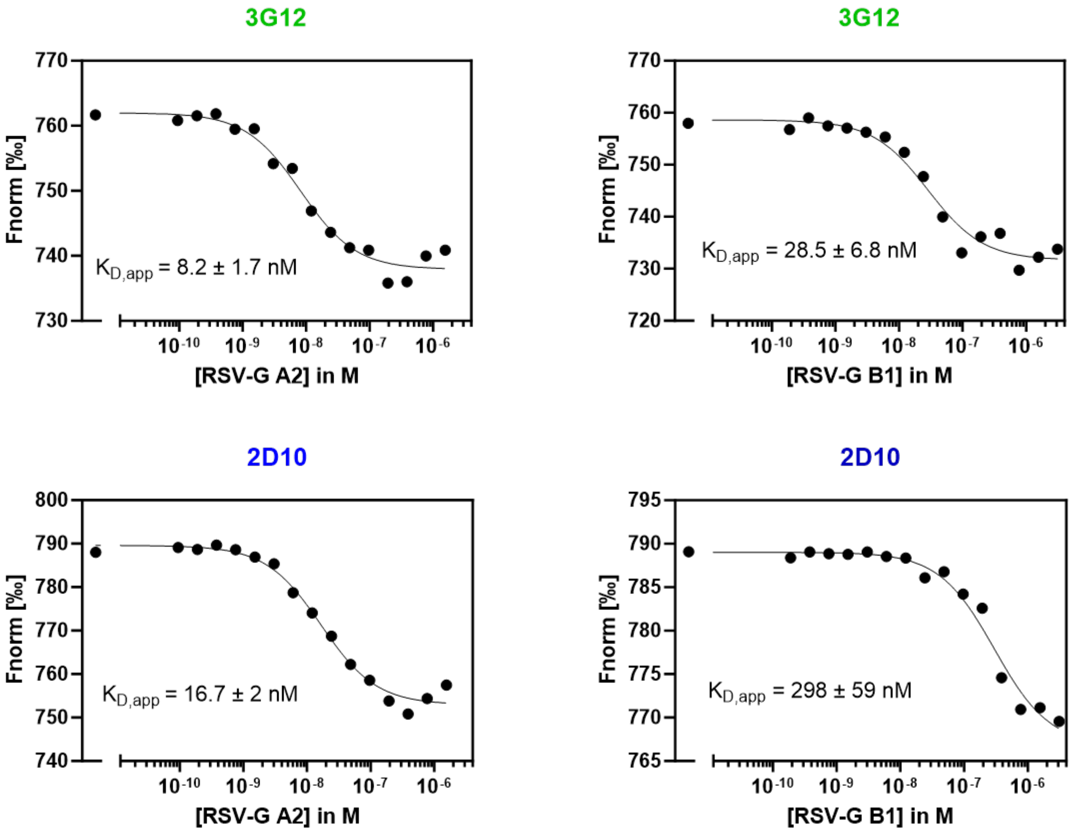

Figure S4.

MST of scFv variants targeting RSV-G

Microscale thermophoresis (MST) experiments for anti-RSV-G scFv versions of 3G12 and 2D10. A final concentration of Alexa647-labelled scFvs of 2 nM was used, with 60% excitation power and 40% MST power. MST on anti-N2 AS4C-HL-DogTag was previously described in (*1*). Shown are traces from single experiments.

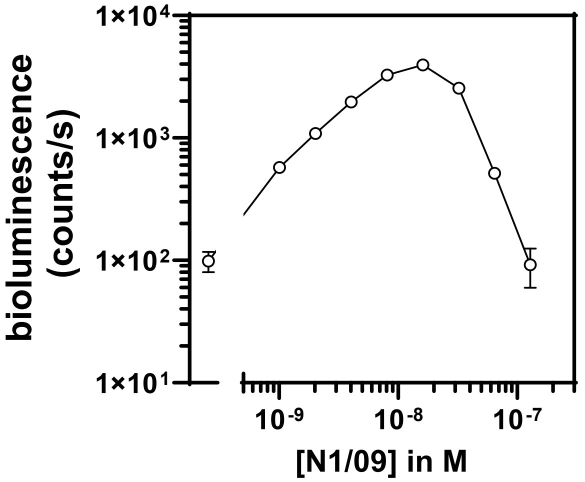

Figure S5.

Monovalent GLOVID with 1GO1 scFv

GLOVID assays with scFv binder 1GO1 conjugated to LgBiT-Dog1 and SmBiT-Dog1, targeting IAV N1/09. Experimental conditions: final GLOVID component concentration 2 nM, 1xPBS + 1 mg/ml BSA, 16 h incubation at 4 °C, final NanoGlo dilution 1:2000. Error bars represent the standard deviation of n=3 replicates.

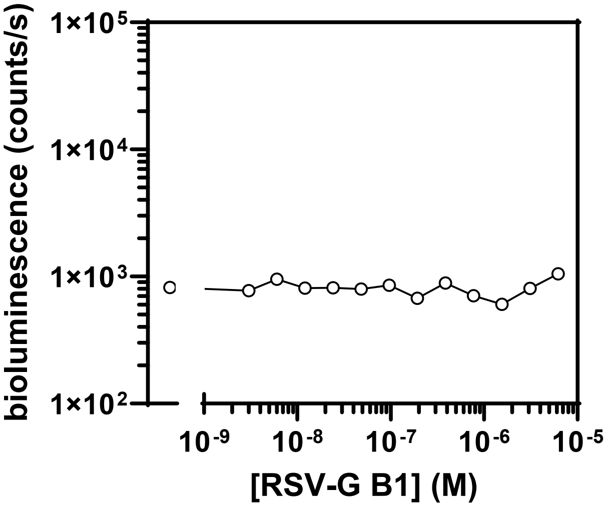

Figure S6.

Controls of anti RSV-G GLOVID (1/2)

GLOVID on RSV-G B1 assay using a combination of 3G12-LgBiT and 2D10-SmBiT. Experimental conditions: 1xPBS plus 1 mg/ml BSA, 2 nM each sensor component, 1 h incubation at 22 °C, 1:2000 diluted NanoGlo substrate. The mean of n=3 replicates is shown, with error bars too small to be depicted.

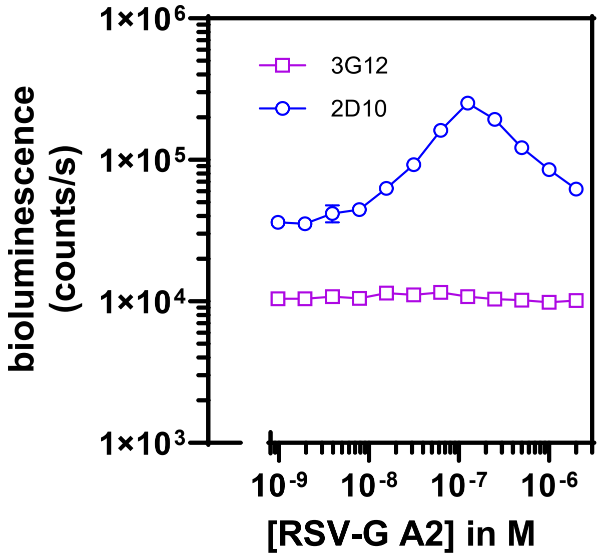

Figure S7.

Controls of anti RSV-G GLOVID (2/2)

GLOVID on RSV-G A2 using the same scFv (3G12 or 2D10) on both sensor components. Experimental conditions: 1xPBS plus 1 mg/ml BSA, 4 nM each sensor component, incubation 4 °C for 16 h to reach full binding equilibrium, 1:2000 diluted NanoGlo substrate. Error bars represent the standard deviation of n=3 replicates.

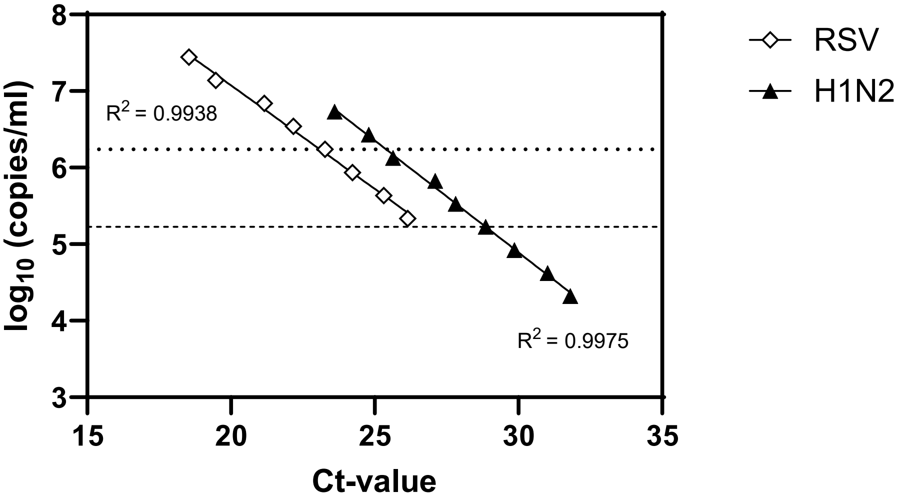

Figure S8.

RT-qPCR standard curves

Standard curves of RT-qPCR on H1N2 (GeneXpert Flu/SARS/RSV triplex plus) and RSV-A2 (Seegene RV essential). The dashed line corresponds to the LoD of the H1N2 GLOVID (Ct 28.86); the dotted line corresponds to the LoD of the RSV-A2 GLOVID (Ct 23.27). Data points represent single measurements from a dilution series of H1N2 or RSV-A2, respectively.

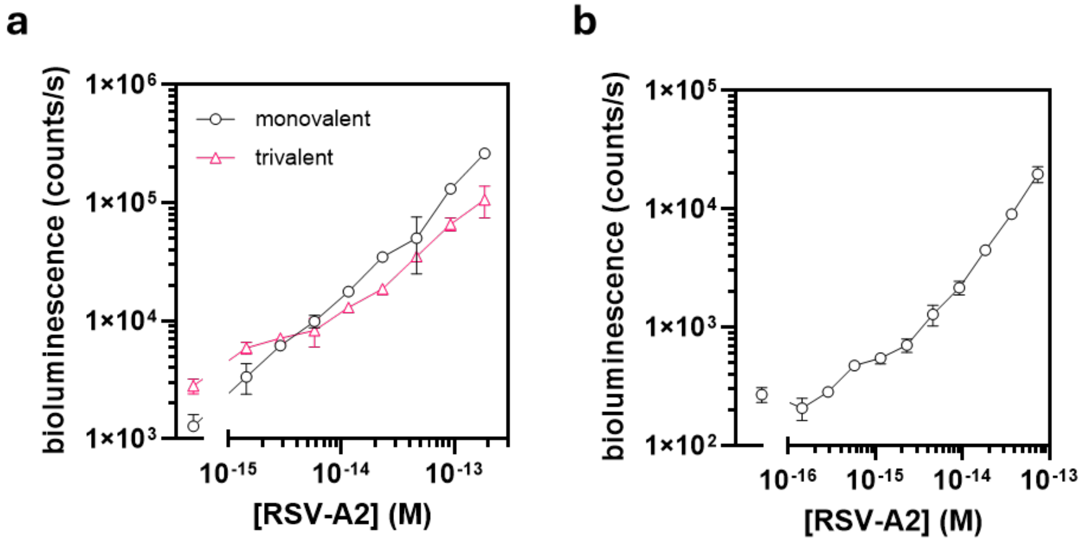

Figure S9.

GLOVID assays targeting RSV-A2 via one surface protein.

a) GLOVID assay that uses F-VHH-4 as binder, in monovalent fashion (F-VHH-4 fused to LgBiT-Dog1 and SmBiT-Dog1) and trivalent fashion (F-VHH-4 fused to LgBiT-Dog3 and SmBiT-Dog3); b) GLOVID assay that uses 3G12 (fused to LgBiT-Dog1) and 2D10 (fused to SmBiT-Dog1) as sensor parts. Experimental conditions: 4 nM final GLOVID component concentration, 1xPBS, final NanoGlo dilution 1:2000, 2 h incubation 22 °C. Error bars represent the standard deviation of n=3 replicates.

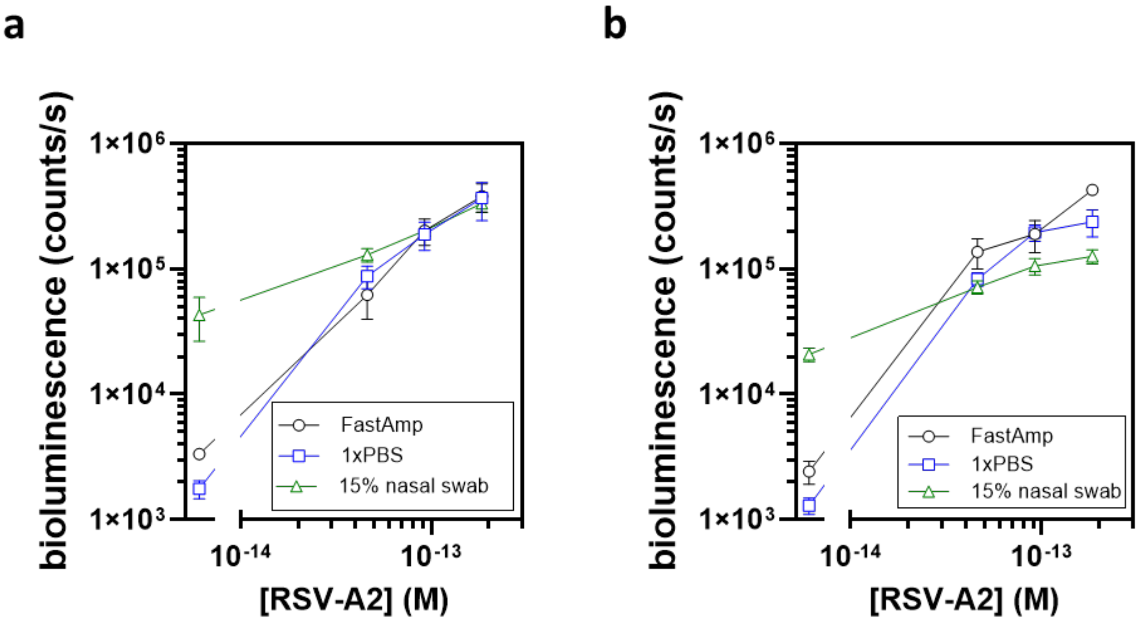

Figure S10.

Spiking experiments in different matrices.

Spiking experiment where virus (RSV-A2) was added to different matrices (FastAmp (Intact Genomics), 1xPBS, 15% diluted nasal swab) and tested with GLOVID via F-VHH-4-LgBiT / F-VHH-4-SmBiT (a) or 2D10-LgBiT / F-VHH-4-SmBiT (b). Experimental conditions: 3 nM LgBiT, 6 nM SmBiT, buffer as indicated in the legend, final NanoGlo dilution 1:1000, 1 h incubation at 22 °C. Error bars represent the standard deviation of n=3 replicates.

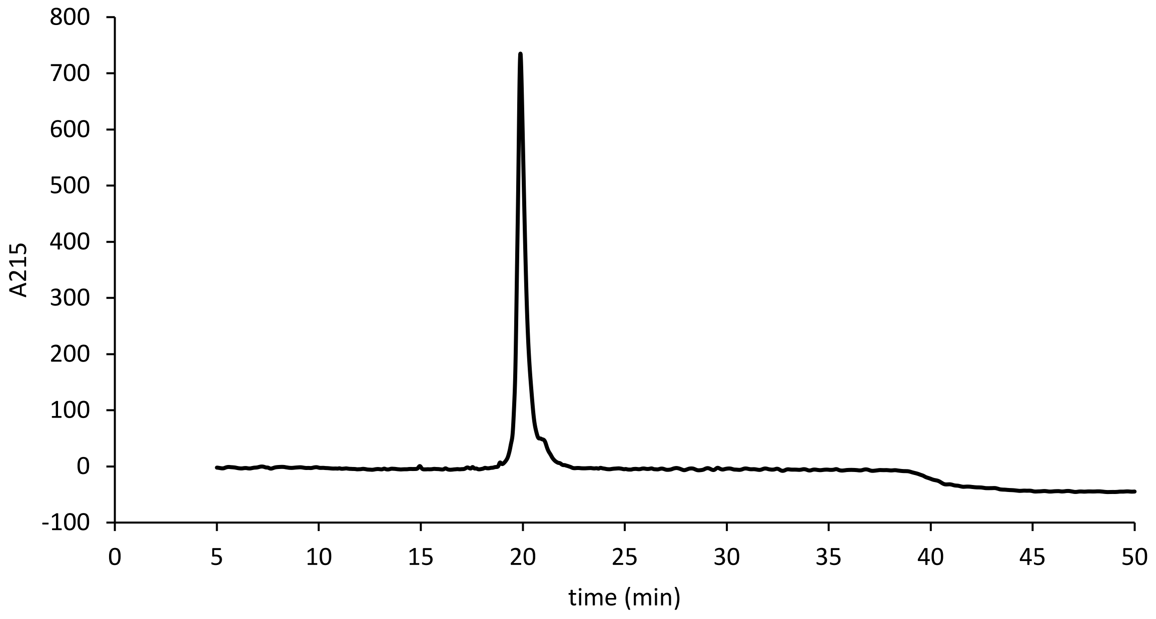

Figure S11.

Analytical HPLC trace of purified final S5-dog tag product.

The purification was performed on a C18 column, with an elution gradient 27.5-47.5% v/v acetonitrile in water with 0.1% trifluoroacetic acid.

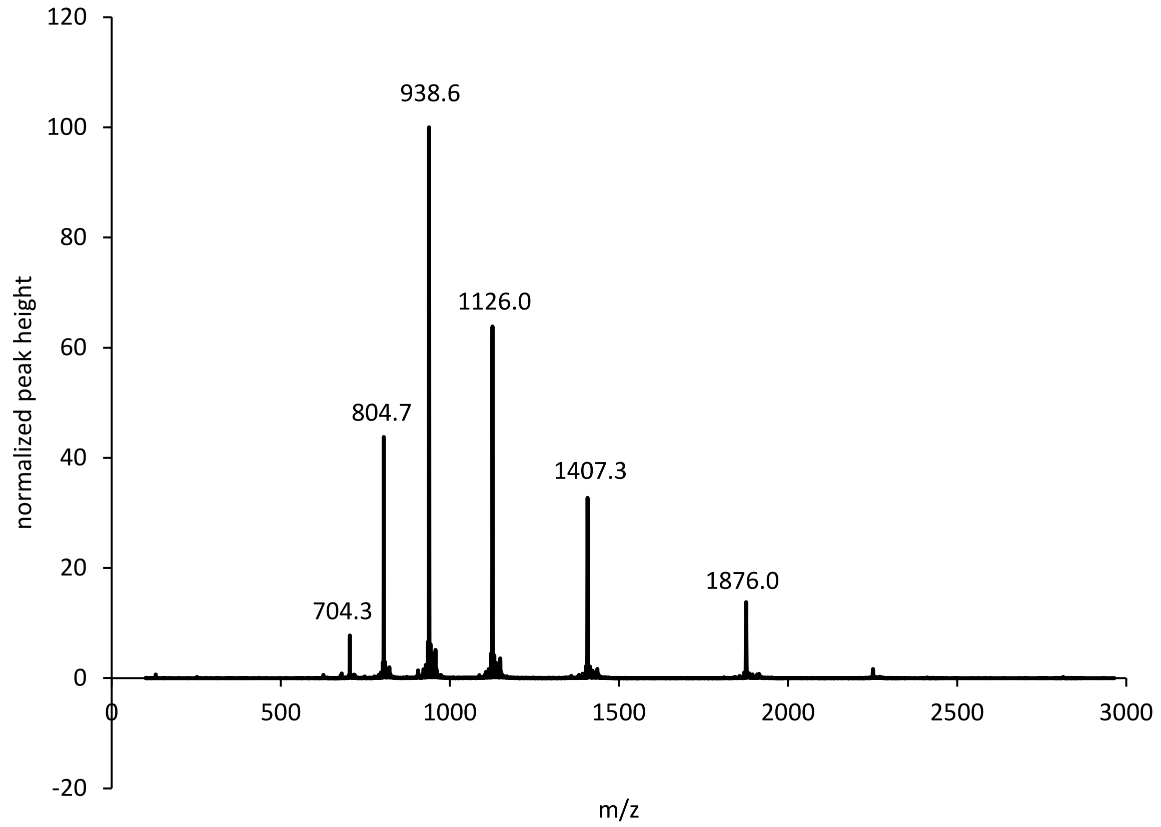

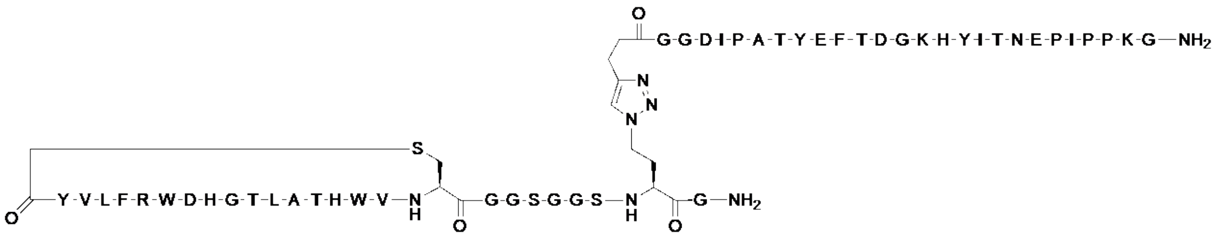

Figure S12.

Mass spectrum of HPLC peak at retention time 19.9 min (Figure S13).

Calculated for final product $C_{255}H_{364}N_{70}O_{74}S$: 5622.66, found 804.7 (calc. for $[M+7H]^{7+}$: 804.2), 938.6 (calc. for $[M+6H]^{6+}$: 938.1), 1126.0 (calc. for $[M+5H]^{5+}$: 1125.5), 1407.3 (calc. for $[M+4H]^{4+}$: 1406.7), 1876.0 (calc. for $[M+3H]^{3+}$: 1875.2).

Section S1 – Supplementary Materials and Methods

Microscale Thermophoresis (MST) experiments

To estimate the K_D of the constructed 2D10-HL-DogTag and 3G12-HL-DogTag scFvs for binding to RSV-G variants, purified scFvs were labeled with Alexa647 and used in MST binding experiments. For labelling, the protein was concentrated using an Amicon filter (10 MWCO) and buffer exchanged to 0.2 M sodium bicarbonate pH 8.3 using PD SpinTrap G-25 columns (Cytiva) according to the manufacturer’s protocol. 100 µl of each protein at ~15 µM was mixed with 10 µl of Alexa-647 NHS Ester (Lumiprobe) freshly dissolved in DMSO (10 mg/ml) and incubated for 1 h at 22 °C with constant shaking. The reaction was purified from excess dyes by subsequently applying it to PD SpinTrap G-25 column twice. The concentration of the protein and the efficiency of labeling was calculated according to (*2*). Labelled scFvs were mixed with varying concentrations of target and incubated for 1 h at 22 °C in a volume of 40 µl in 1xPBS & 0.02% Tween20 (final scFv concentration 2 nM). Capillaries were loaded and MST experiment was performed at 60% LED power and 40% MST power on a Monolith NT.115 (NanoTemper Technologies).

ddPCR

Viral RNA from virus samples was prepared for digital-droplet PCR (ddPCR) by adding an inactivation buffer (200 mM TCEP, 2 mM EDTA, 2 U/µl murine RNase inhibitor, 20 mM Tris-HCl, pH 8.0) to the sample in 1:1 ratio, followed by incubation at 95°C for 5 min. Subsequently, the 1-Step RT-ddPCR Advanced Kit for Probes (BioRad) was used in combination with the CFX96 thermocycler and the QX200 ddPCR system, using the following oligos and probes depending on the virus targeted. Data was analysed using BioRad QX One (v1.2). The oligos target the M gene of IAV and the M gene of RSV, respectively.

ddPCR oligo list

IAV targeting oligos	sequences (5’ → 3’)
FLUAM-7-F	CTTCTAACCGAGGTCGAAACGTA
FLUAM-161-R	GGTGACAAGATTGGTCTTGTCTTTA
FLUAM-49-P	TCAGGCCCCCTCAAAGCCGAG
RSV targeting oligos	
RSVM-F	GGCAAATATGGAAACATACGTGAA
RSVM-R	TCTTTTTCTAGGACATTGTATTGAACAG
RSVM-P	CTGTGTATGTGGAGCCTTCGTGAAG

Influenza A virus and RSV sample collection from residual clinical materials

Patients were swabbed with eSwab (Copan, Italy) flocked tips, containing 1 ml of liquid modified Amies fluid. Molecular diagnostics were performed using GeneXpert SARS-CoV-2/Flu/RSV rapid test (Cepheid, Sunnyvale, CA, USA), BioFire RP2.1plus rapid test (bioMérieux, France), and Allplex RV Essential assay RT-PCR (Seegene, Seoul, South Korea) combined with the FlowGO middle ware (LabHelp Labautomation, Bladel, the Netherlands) as previously described (*3*). Standard curves for correlating the ddRT-PCR experiments to GeneExpert and Seegene Ct-values were obtained from dilution series of the A/swine/Italy/114922/2014 (H1N2) and RSV-A2 stocks.

Section S2

Protein sequences

The used tags for protein purifications are hexa-His-tag (HHHHHH) and Strep-tag II (WSHPQFEK).

LgBiT-DogCatcher (LgBiT-Dog1):
Blue: LgBiT, Red: DogCatcher
MGTSVFTLEDFVGDWEQTAAYNLDQVLEQGGVSSLLQNLAVSVTPIQRIVRSGENALKIDIHVIIPYEGLSADQMAQIEEVFKVVYPVDDHHFKVILPYGTLVIDGVTPNMLNYFGRPYEGIAVFDGKKITVTGTLWNGNKIIDERLITPDGSMLFRVTINSSGGGTKLGEIEFIKVDKTDKKPLRGAVFSLQKQHPDYPDIYGAIDQNGTYQDVRTGEDGKLTFTNLSDGKYRLIENSEPPGYKPVQNKPIVSFRIVDGEVRDVTSIVPQGGGSWSHPQFEK*

DogCatcher-SmBiT (SmBiT-Dog1):
Red: DogCatcher, yellow: SmBiT101 (*4*)
MGTKLGEIEFIKVDKTDKKPLRGAVFSLQKQHPDYPDIYGAIDQNGTYQDVRTGEDGKLTFTNLSDGKYRLIENSEPPGYKPVQNKPIVSFRIVDGEVRDVTSIVPQGKLGGSGGSGGSGGSGGSGGSGGSGGSGGENLYFQSGGSGGSGGSGGSGGSGGSGGSGGTGSVTGYRLFEKESGGSGGSWSHPQFEK*

SD36-DogTag:
Blue: SD36, green: DogTag.
MGSVQLVESGGGLVQAGGSLKLSCAASGRTYAMGWFRQAPGKEREFVAHINALGTRTYYSDSVKGRFTISRDNAKNTEYLEMNNLKPEDTAVYYCTAQGQWRAAPVAVAAEYEFWGQGTQVTVSGGSGGSGTGDIPATYEFTDGKHYITNEPIPPKGGSGGSWSHPQFEK*

HSB2.A-DogTag:
Blue: HSB2.A, green: DogTag.
MGSHHHHHHSSGGIVNVPNCNTTKYQQLARTAVAIYNYHEQAHLTFVENLNCKEQGNYYYITLAATDDAGKKAIYEAKIGVVESAGWTGVEEFKLVGSGGSGGSGGSGGSGGSSGGSGSGTGDIPATYEFTDGKHYITNEPIPPKGGSGGSWSHPQFEK*

SD38-DogTag:
Blue: SD38, green: DogTag.
MEVQLVESGGGLVQPGGSLRLSCAVSISIFDIYAMDWYRQAPGKQRDLVATSFRDGSTNYADSVKGRFTISRDNAKNTLYLQMNSLKPEDTAVYLCHVSLYRDPLGVAGGMGVYWGKGALVTVSSKLGGSGGSGGSGGSGGSTGDIPATYEFTDGKHYITNEPIPPKGGSGGSWSHPQFEK*

F-VHH-4-DogTag:
Blue: F-VHH-4, green: DogTag
MGSQVQLQESGGGLVQPGGSLRLSCAASGFTLDYYYIGWFRQAPGKEREAVSCISGSSGSTYYPDSVKGRFTISRDNAKNTVYLQMNSLKPEDTAVYYCATIRSSSWGGCVHYGMDYWGKGTQVTVSSKLGGSGGSGGSGGSGGSTGDIPATYEFTDGKHYITNEPIPPKGGSGGSWSHPQFEK*

LgBiT-3xDogCatcher (LgBiT-Dog3)
Blue: LgBiT, red: DogCatcher (3 times)

MGTSVFTLEDFVGDWEQTAAYNLDQVLEQGGVSSLLQNLAVSVTPIQRIVRSGENALKIDIHVIIPYEGLSADQMAQIEEVFKVVYPVDDHHFKVILPYGTLVIDGVTPNMLNYFGRPYEGIAVFDGKKITVTGTLWNGNKIIDERLITPDGSMLFRVTINGGSGELTGGSGGSGGSGGSGGSGGSGEFAEAAAKEAAAKEAAAKEAAAKEAAAKEAAAKAEFGGSGGSGGSGGSGGSGGSGGTKLGEIEFIKVDKTDKKPLRGAVFSLQKQHPDYPDIYGAIDQNGTYQDVRTGEDGKLTFTNLSDGKYRLIENSEPPGYKPVQNKPIVSFRIVDGEVRDVTSIVPQTSTPTPTPTPTPTPTPTPTPTPTPTPTPTPTPTPTPTPTPTPTPTPTPASKLGEIEFIKVDKTDKKPLRGAVFSLQKQHPDYPDIYGAIDQNGTYQDVRTGEDGKLTFTNLSDGKYRLIENSEPPGYKPVQNKPIVSFRIVDGEVRDVTSIVPQTGPTPTPTPTPTPTPTPTPTPTPTPTPTPTPTPTPTPTPTPTPTPKLGEIEFIKVDKTDKKPLRGAVFSLQKQHPDYPDIYGAIDQNGTYQDVRTGEDGKLTFTNLSDGKYRLIENSEPPGYKPVQNKPIVSFRIVDGEVRDVTSIVPQGGGSWSHPQFEK*

3xDogCatcher-SmBiT (SmBiT-Dog3):
Red: DogCatcher (3 times), yellow: SmBiT101
MGTKLGEIEFIKVDKTDKKPLRGAVFSLQKQHPDYPDIYGAIDQNGTYQDVRTGEDGKLTFTNLSDGKYRLIENSEPPGYKPVQNKPIVSFRIVDGEVRDVTSIVPQTSTPTPTPTPTPTPTPTPTPTPTPTPTPTPTPTPTPTPTPTPTPTPASKLGEIEFIKVDKTDKKPLRGAVFSLQKQHPDYPDIYGAIDQNGTYQDVRTGEDGKLTFTNLSDGKYRLIENSEPPGYKPVQNKPIVSFRIVDGEVRDVTSIVPQTGPTPTPTPTPTPTPTPTPTPTPTPTPTPTPTPTPTPTPTPTPTPKLGEIEFIKVDKTDKKPLRGAVFSLQKQHPDYPDIYGAIDQNGTYQDVRTGEDGKLTFTNLSDGKYRLIENSEPPGYKPVQNKPIVSFRIVDGEVRDVTSIVPQGGGSGELTGGSGGSGGSGGSGGSGGSGEFAEAAAKEAAAKEAAAKEAAAKEAAAKEAAAKAEFGGSGGSGGSGGSGGSGGSGGTVTGYRLFEKESGGSHHHHHH*

scFvs:

scFv sequences are listed in their secreted form, omitting the α-factor secretion signal present in pPICZalphaB.

AS4C-HL-DogTag:
The original heavy and light chain sequences of the AS4C antibody were provided by Pramila Rijal and Alan Townsend. A heavy chain-light chain (HL) configuration scFv was designed based on these sequences.
Green: DogTag
EAEAAG(*AS4C_heavy_chain*)GGGGSGGGGSGGGGS(*AS4C_light_chain*)GTSGGGSGTGDIPATYEFTDGKHYITNEPIPPKGGSGGSHHHHHH*

1GO1-HL-DogTag:
Underlined: 1GO1-HL, green: DogTag
EAEAAGEVQLVESGGRALRPGGSLRLSCAASGFKFDDYAMSWVRQVPGKGLEFVSGLNWNGDITAYTDSVKGRFTVSRDNAKNSLYLHINSPKPEDTALYYCARTSSWGDYTRGPEPKITWYFDLWGRGTLVTVSSGGGGSGGGGSGGGGSDIQLTQSPSFLSASVGDRITITCRASQGIDGYLAWYQQRPGKAPNLLIYAASLLQSGVPSRFSGSGYGTEFTLTISSLQPEDFATYYCQHLDSYPLFTFGPGTKVDIKRTGTSGGGSGTGDIPATYEFTDGKHYITNEPIPPKGGSGGSHHHHHH*

2D10-HL-DogTag:
Underlined: 2D10-HL, green: DogTag
EAEAAGQVQLVQSGPEVKKPGASVRLSCKASGYVFTNYGVSWVRQAPGQGLEWMGWSSPYNGNTYYAQKLKARVTMTTDTSTNTAYMELRSLRSDDTAVYYCGRDMLGVVQAVAGPFDSWGQGTLVTVSSASGGGGSGGGGSGGGGSGGGDTPMTQSPSSVSASVGDRVTISC

RASQGISNSLAWYQQKLGKAPQLLIYAASSLQSGVPSRFSGSGSGTDFTLTISSLQPEDFATYYCQQTNTFPFTFGPGTKVEVRRGTSGGGSGTGDIPATYEFTDGKHYITNEPIPPKGGSGGSHHHHHH*

3G12-HL-DogTag

Underlined: 3G12-HL, green: DogTag

EAEAAGQLQLQESGPGLVKPSETLSLTCTVSGGSISSSNYYWGWIRQPPGKGLEWIASIHDSGSIYYNPSLRSRVTISVDTSKNQFSLKLSSVTAADTAVYYCARHLVWFGELRNNWFDPWGQGTLVTVASGGGGSGGGGSGGGGSGGGEIVMTQSPATLSVSPGERATLSCRASQSVNSNLAWYQHKPGQAPRLLIYGASTRATGIPARFSGSGSGTDFTLTISSLQSEDFAVYYCQQYNNWPLFGPGTKVDLKRTGTSGGGSGTGDIPATYEFTDGKHYITNEPIPPKGGSGGSHHHHHH*

References

1. A. Gräwe, C. M. Spruit, R. P. de Vries, M. Merkx, Bioluminescent detection of viral surface proteins using branched multivalent protein switches. *RSC Chem Biol* **5**, 148–157 (2024).
2. J. S. Nanda, J. R. Lorsch, "Labeling a Protein with Fluorophores Using NHS Ester Derivitization" in *Methods in Enzymology*, J. R. Lorsch, Ed. (2014) vol. 536, pp. 87–94.
3. J. Flipse, A. T. Tromp, D. Thijssen, N. van Xanten-Jans-Beken, R. Pauwelsen, H. J. van der Veer, J. M. Schlaghecke, C. M. A. Swanink, Optimization of the STARlet workflow for semi-automatic SARS-CoV-2 screening of swabs and deep respiratory materials using the RealAccurate Quadruplex SARS-CoV-2 PCR kit and Allplex SARS-CoV-2 PCR kit. *Microbiol Spectr* **12** (2024).
4. A. S. Dixon, M. K. Schwinn, M. P. Hall, K. Zimmerman, P. Otto, T. H. Lubben, B. L. Butler, B. F. Binkowski, T. MacHleidt, T. A. Kirkland, M. G. Wood, C. T. Eggers, L. P. Encell, K. V. Wood, NanoLuc Complementation Reporter Optimized for Accurate Measurement of Protein Interactions in Cells. *ACS Chem Biol* **11**, 400–408 (2016).
